# Accurate Real-Time Live Face Detection Using Snapshot Spectral Imaging Method

**DOI:** 10.3390/s25030952

**Published:** 2025-02-05

**Authors:** Zhihai Wang, Shuai Wang, Weixing Yu, Bo Gao, Chenxi Li, Tianxin Wang

**Affiliations:** 1 Key Laboratory of Spectral Imaging Technology, Xi’an Institute of Optics and Precision Mechanics, Chinese Academy of Sciences, Xi’an 710119, China; wangzhihai@opt.ac.cn (Z.W.); gaobo_101@opt.ac.cn (B.G.); lichenxi@opt.ac.cn (C.L.); wangtianxin@opt.ac.cn (T.W.); 2 Center of Mechanics Science and Optoelectronics Engineering, University of Chinese Academy of Sciences, Beijing 101408, China

**Keywords:** spectral image, live face detection, neural network

## Abstract

Traditional facial recognition is realized by facial recognition algorithms based on 2D or 3D digital images and has been well developed and has found wide applications in areas related to identification verification. In this work, we propose a novel live face detection (LFD) method by utilizing snapshot spectral imaging technology, which takes advantage of the distinctive reflected spectra from human faces. By employing a computational spectral reconstruction algorithm based on Tikhonov regularization, a rapid and precise spectral reconstruction with a fidelity of over 99% for the color checkers and various types of “face” samples has been achieved. The flat face areas were extracted exactly from the “face” images with Dlib face detection and Euclidean distance selection algorithms. A large quantity of spectra were rapidly reconstructed from the selected areas and compiled into an extensive database. The convolutional neural network model trained on this database demonstrates an excellent capability for predicting different types of “faces” with an accuracy exceeding 98%, and, according to a series of evaluations, the system’s detection time consistently remained under one second, much faster than other spectral imaging LFD methods. Moreover, a pixel-level liveness detection test system is developed and a LFD experiment shows good agreement with theoretical results, which demonstrates the potential of our method to be applied in other recognition fields. The superior performance and compatibility of our method provide an alternative solution for accurate, highly integrated video LFD applications.

## 1. Introduction

Facial recognition technology, a form of biometric technology, is based on the identification of facial characteristics [1,2] and has been well developed and has found wide applications in areas related to identification verification, such as secure access control, smart surveillance, facial payments, etc. Obviously, the systems just mentioned above are all closely associated with security, which poses higher requirements for the accuracy rate and anti-spoofing ability of face recognition. However, traditional visual-based systems that solely rely on two-dimensional (2D) facial images are vulnerable to various spoofing techniques, such as printed photographs or human replica face masks [3,4]. Consequently, face anti-spoofing plays a critical role in the field of biometric verification, aiming to accurately distinguish between genuine human faces and counterfeit ones. However, with the development of 3D printing and biomimetic silicone technology, 3D face masks and models have become a more effective way to attack traditional live face detection (LFD) technology based on RGB image analysis [5] or video analysis [6]. To address this issue, the incorporation of advanced techniques that offer additional information beyond visual data is necessary for live face detection (LFD) [7,8]. Recently, spectral imaging technology has emerged as a promising solution for LFD by capturing and analyzing the unique spectral reflectance properties of facial material [9]. The reflectance spectra of living human skin exhibit different characteristics due to the difference in the absorption spectrum of skin and hemoglobin in human blood [10,11]. By detecting the human skin reflectance spectrum, we can effectively distinguish between real and fake faces. Furthermore, spectral imaging systems possess the unique ability to capture 2D spatial information and spectral dimension information, enabling both face recognition and LFD simultaneously [12]. Traditional spectral imaging technology needs to obtain multi-spectral images by scanning. To reduce scanning time, researchers had to use only a few spectral channels to build an LFD system [13,14], which performed badly in inter-dataset evaluations [15]. Among the new spectral imaging systems, snapshot spectral imaging stands out due to its instantaneous scanning capability, which perfectly aligns with LFD applications [15]. With advancements in semiconductor processing, micro-nano processing, and deep neural network technologies, snapshot spectral CMOS image sensors (CISs) have significantly enhanced system integration and stability while substantially reducing costs [16,17,18,19], and hyperspectral data can be generated by a single sensor [20,21]. They have emerged as valuable tools within the field of LFD [22].

In our work, we designed a novel real-time LFD method utilizing a compact spectral CIS and built a system based on this method. The key characteristics of this system include compact size, high accuracy, and seamless integration. We employ computational spectral imaging technology to achieve precise spectral reconstruction and enable real-time liveness detection. The maximum frame rate for capturing snapshots of our system is 30 frames per second (fps), eliminating the need for time-consuming scanning procedures. Compared to traditional spectral LFD approaches that require scanning to acquire spectra, our system can capture facial images in real-time, making it fully compatible with real-time dynamic face identification usage scenarios. Meanwhile, rapid detection based on hyperspectral data also brings excellent detection accuracy. Moreover, in contrast to existing snapshot hyperspectral LFD approaches [15] that rely on detecting fixed-angle faces and specified points of the face to extract spectra, our method enables automatic selection of flat facial skin areas and rapid point-by-point spectral reconstruction based on Tikhonov regularization theory [23]. This capability demonstrates its effectiveness in multi-pose face liveness detection. In addition, we selected the convolutional neural network (CNN) as a face classifier because CNNs demonstrate outstanding capabilities in extracting features, particularly in the aspect of automatically extracting spectral features in spectral data. Ultimately, the huge database acquired through the rapid point-by-point spectral reconstruction algorithm and the trained CNN model contribute to high detection accuracy. This innovative approach effectively mitigates the barriers to adopting LFD systems based on spectral imaging, thereby providing an alternative solution for highly integrated and accurate video LFD applications.

## 2. Related Work

The original facial recognition technology relied on RGB cameras, which was a convenient and fast biometric verification method that was widely used. Given that 2D images were commonly used as facial spoofing at that time, the LFD method based on RGB images rapidly gained popularity. They primarily achieve LFD by relying on the color distribution features of images, through manual feature extraction, such as SIFT [24], HOG [25], etc. These methods usually require specific prior features and are easily crackable. With the advancement of neural networks, people are capable of conducting LFD by employing more complex features, such as facial contours, gradients, texture details, etc. Among these, the 2D CNN is the most mature one [26]. However, with the development of facial recognition technology, more high-value information employs facial information as an encryption approach, accompanied by the emergence of more high-cost facial spoofing techniques, such as face masks and face models. The RGB camera is capable of collecting only limited information, and it is difficult to accurately detect spoofing based on outlines, edges, etc. Therefore, LFD requires a camera to have the ability to capture multi-dimensional information. This difficulty can be mitigated to some extent by additional sensors, such as RGBD and NIR, but the small amount of information on extra dimensions obtained from added sensors is not sufficient to support feature selection, and it makes them perform badly on inter-dataset evaluations [27]. Meanwhile, some advanced sensors, including SWIR [28], thermal cameras [29], and polarization cameras [30], are used to analyze and detect the skin features of real and fake faces, achieving higher detection accuracy. However, they are either too large, too complex, or too expensive, making it difficult to integrate them with real-world face recognition systems.

Current face spoofing encompasses silicone or synthetic resin model faces, face masks, color printed pictures, etc. In contrast, facial skin is a complex multi-layered tissue and has three main layers, which are the epidermis, dermis, and hypodermis [31]. It means that facial skin possesses distinctive spectral characteristics that can be used to distinguish real and fake faces. The reflectance spectra of living human skin exhibit different characteristics due to the difference in the absorption spectrum of skin and hemoglobin in human blood [10,11]. Spectral imaging systems possess the unique ability to capture 2D spatial information and spectral dimension information, enabling face recognition and spectral detection simultaneously [12]. The outcome of spectral imaging is three-dimensional data, which includes two-dimensional spatial data and one-dimensional spectral data. Traditional hyper-spectral imaging systems (HSIs) [11] are capable of detecting up to two dimensions per exposure only, which requires multiple scans for comprehensive coverage. Consequently, they possess drawbacks such as being expensive and bulky and requiring scanning, which also makes it arduous to integrate them with real-world face recognition systems.

In recent years, the emergence of snapshot spectral imaging cameras has significantly ameliorated these issues. They employ micro-nanostructures, such as metasurfaces or photonic crystals, to fabricate miniature filters that cover the surface of each pixel, making the spectral camera as compact as a conventional RGB camera. On the one hand, these cameras possess the capability of capturing real-time images, which can be used to identify identities by the face recognition system, which has already reached a high level of maturity. On the other hand, the camera can capture the spectra of each pixel in real time, which can be used to detect the authenticity of the face, significantly enhancing the anti-spoofing capability of the face recognition system. Nevertheless, they still present issues such as narrow working spectral bandwidth or severe channel crosstalk problems, which require algorithm optimization and LFD system design [16,17,18,19,20,21,22].

## 3. Design and Principle

### 3.1. Working Principle

**Figure 1:**
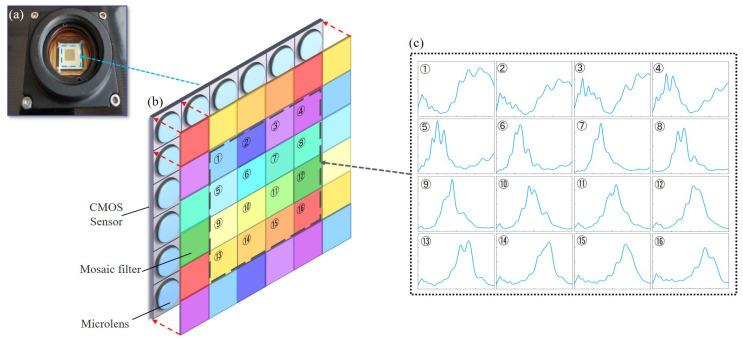
Structure and principle of the snapshot spectral sensor. (**a**) A snapshot spectral CIS camera with 1920 × 1080 pixels. (**b**) Schematic diagram of the mosaic filter structure of a sensor with 16 channels. The CMOS surface is initially attached to a layer of pixel-level micro-lenses and subsequently to a layer of spectral filter arrays. The micro-lenses are employed to enhance the light-gathering capacity of the sensor, while the filters constitute a 4 × 4 spectral filter array arranged in a periodic mosaic pattern. The red dot arrow in (**b**) shows that the micro-lens and filter are the same size as each pixel and fit perfectly. Each 4 × 4 pixel block, containing 16 different filters, constitutes a filter unit and the pixel block in the black dot frame is one of them. (**c**) The spectral responses of 16 spectral channels in a filter unit.

The spectral features of human skin, particularly in the range of 500–650 nm, are essential for accurate liveness detection [11]. For the sake of capturing the human skin spectra accurately, we selected a 16-channel customized snapshot spectral image sensor that serves as the spectral sensing module for our LFD system. As shown in Figure 1a,b, the CMOS detector surface incorporates 4 × 4 spectral filter arrays that are arranged in a periodic mosaic pattern, resembling the RGGB Bayer pattern [32]. Each filter channel is precisely aligned with the CMOS detector pixel, the channel is the same size as the pixel, and they fit perfectly. As shown in Figure 1c, each filter unit is comprised of 16 channels with distinct peak wavelengths of the transmission spectra, which allows for recovery of the incident spectra based on different spectral responses. By capturing adjustable monochromatic light processed through an integrating sphere, the spectral response of each spectral channel can be obtained. We utilized a method of multiple captures followed by averaging, effectively reducing spectral response error caused by CIS noise. At present, the transmission principles of current Bayer-like CIS are largely consistent, with the primary difference being the spectral responses; thus, they face similar challenges, including spectral crosstalk and complex and time-consuming algorithms, among others [15,16,17,19]. Therefore, the method we present below offers compatibility across various Bayer-like spectral imaging systems.

**Figure 2:**
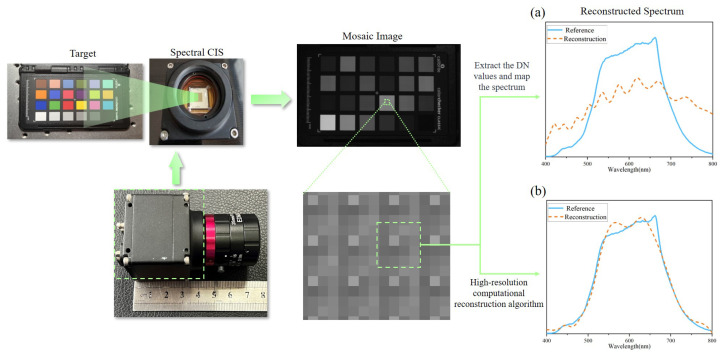
The overall architecture of the snapshot spectral CIS captured and acquired the target spectrum. Each set of 16 channels in mosaic images is viewed as a period, and their DN values can be reconstructed as a spectrum. Unlike traditional spectral filters, spectral crosstalk exists in micro-filter arrays. (**a**) The spectral resolution acquired by employing the traditional DN weighting method is extremely low. (**b**) The utilization of computational spectral reconstruction algorithms can conspicuously elevate spectral resolution.

The CMOS sensor has a spatial resolution of 1920 × 1080 pixels. Traditional face recognition relies mainly on cameras that operate in the visible spectrum. To ensure that spectral cameras are compatible with such technologies, it is necessary to capture visible light information within the 380–780 nm range more comprehensively while excluding the boundary of the visible spectrum, where the quantum efficiency of our CMOS is relatively low. As a result, the 400–750 nm range was ultimately selected as the detection spectral band, and a 400–750 nm bandpass filter was integrated into the optical lens. Each filter unit in the sensor can be seen as a miniature spectrometer for visible light. As illustrated in Figure 2, the spectral CIS captured a mosaic image of a color card in a single shot. The DN values assigned to each channel, detected by the “micro-spectrometer”, contain unique spectral response information, which can be reconstructed from the spectrum. Traditional filters that are ample in size to cover the CMOS surface can possess high spectral resolution and extremely low spectral crosstalk, enabling the direct mapping of DN values to the target spectrum *F*:

$$F\approx \sum_{i}^{16}I_{i}\cdot T_{i}$$

where $I_{i}\left(i=1,2,3,\cdots ,16\right)$ is the DN values of each channel. $T_{i}\left(i=1,2,3,\cdots ,16\right)$ denotes the spectral response of each channel. However, as shown in Figure 2a, the crosstalk between different channels among micro-filters is capable of significantly reducing the spectral resolution of traditional reconstruction approaches. Consequently, computational spectral reconstruction algorithms possessing the capabilities to resolve spectral crosstalk are required. Currently, typical computational spectral reconstruction algorithms are time-consuming, requiring several hours to reconstruct all data points for a single face [22]. And for that we developed a computational spectral reconstruction algorithm based on Tikhonov regularization theory [33], which is able to convert the captured multi-spectral mosaic image into a hyperspectral image cube rapidly and precisely. The reconstruction principle will be explained in more detail in the following.

The acquisition of spectral information relies on distinct spectral channels. The relationship between the DN value *I* collected by the detector in each period in one shot and the incident target spectra *F* can be expressed as follows:

(1) $$I_{i}=\int F\left(\lambda \right)Q\left(\lambda \right)T_{i}\left(\lambda \right)d\lambda$$

where *Q* represents the quantum efficiency of the sensor, and $I_{i}\left(i=1,2,3,\cdots ,16\right)$ is the DN values of each channel. $T_{i}\left(i=1,2,3,\cdots ,16\right)$ denotes the spectral response of each channel. In discrete form, the equation can be presented as:

(2) $$I_{i}=\sum f\left(\lambda \right)q\left(\lambda \right)t_{i}\left(\lambda \right)$$

where *f*, *q*, and $t_{i}$ are the discrete forms of *F*, *Q*, and $T_{i}$. Notably, the quantum efficiency *q* and spectral response *t* are irrelevant, allowing us to simplify $m_{i}\left(\lambda \right)=q\left(\lambda \right)t_{i}\left(\lambda \right)\left(i=1,2,3,\cdots ,16\right)$. Converted to matrix form, I can be expressed as

(3) I=Mf

where $I=(I_{1},I_{2},I_{3},\cdots )$, $M=(M_{1};M_{2};M_{3};\cdots )$. Obviously, the length of target matrix *f* is significantly larger than the given matrix *I*, suggesting that the matrix is ill-conditioned. In such cases, we need to solve it using Tikhonov regularization theory. This involves transforming the matrix equation into an optimization expression as show below: [33]:

(4) $$\min_{f}{∥Mf-I∥}_{2}$$

This objective function indicates the search for *f* such that the 2-norm of the residual r=Mf−I of the linear equation system *Mf* = *I* is minimized. Nevertheless, such minimization might still result in unstable solutions due to the ill-conditioned nature of the matrix M. Our system inevitably suffers from noise originating from CMOS sensors, calibration processes, and image processing algorithms; finally, approximately 4% error exists in DN value of each pixel. This error is amplified in the ill-conditioned matrix, leading to spectral reconstruction distortions. Hence, further regularization is required [23]:

(5) $$\min_{f}\left\{{∥Mf-I∥}_{2}^{2}+\alpha \cdot {∥f∥}_{2}^{2}\right\}$$

where α is the regularization coefficient, which is capable of reducing the spectral reconstruction resolution while simultaneously minimizing the noise, enhancing robustness. The LFD system requires high spectral fidelity rather than high spectral resolution. Adaptive adjustment algorithms such as GCV and L-curve provide limited improvements in spectral fidelity while consuming a significant amount of computational power. Therefore, a relatively high regularization coefficient α can be maintained to rapidly handle different facial spectral reconstructions. As long as the face being measured is not under extremely-low-illumination conditions, significant adjustments to α are unnecessary. The square of the 2-norm is a convex function, ensuring that the optimization problem with the regularization term appended belongs to the category of convex optimization problems and always guarantees the existence of the global optimal solution. Since in the case of liveness detection, where fixed active light sources are predominantly used in the same environment, the reconstruction process can be transformed into an optimization expression, which reduces the impact of the ill-conditioned equations, mitigating the issues of overfitting and distortion [34].

Matrix *M* can be decomposed into a product of the orthogonal matrix and diagonal matrix through singular value decomposition (SVD) [35]:

(6) $$M=USV^{T}$$

According to the nature of the matrix transformation [36], order $f_{0}=V^{T}f$, we convert the optimization expression obtained in step (4) into

(7) $$\min_{f}\left\{{∥USf_{0}-I∥}_{2}^{2}+\alpha \cdot {∥Vf_{0}∥}_{2}^{2}\right\}$$

In order S=s·b, *s* is a diagonal vector and *b* is a rectangular identity matrix. Solving the quadratic simplification and optimization expression yields the approximate solution for the target spectrum [37]:

(8) $$f=\left(Vb\right)\left(s\cdot \left(U^{T}I\right)\right)/\left(s^{2}+\alpha \right)$$

In our study, we implemented a bandpass filter with a wavelength range of 400–750 nm in front of the camera to eliminate stray light outside the working band. Subsequently, we utilized a monochromator to calibrate the CIS spectra response *M*, with a selected sampling interval of 2 nm, resulting in 176 sampling points. The length of matrix *M* and *f* is 176. Each spectrometer consists of 16 channels, and these channels contribute their respective DN values to construct the input matrix *I*.

### 3.2. Data Processing

Spectral imaging based on real objects must account for the irregularities of the edges of objects and the inhomogeneity of reflectance [38]. This leads to variations in spectral information collected by the spectral channel, which presents challenges for accurate spectral reconstruction. A pixel could initially only capture the DN value of one channel; however, in situations where spectra are gathered from uneven objects or under uneven lighting conditions across different channels, there can be variations in the environment or object being captured. These variations introduce errors in the measured data.

**Figure 3:**
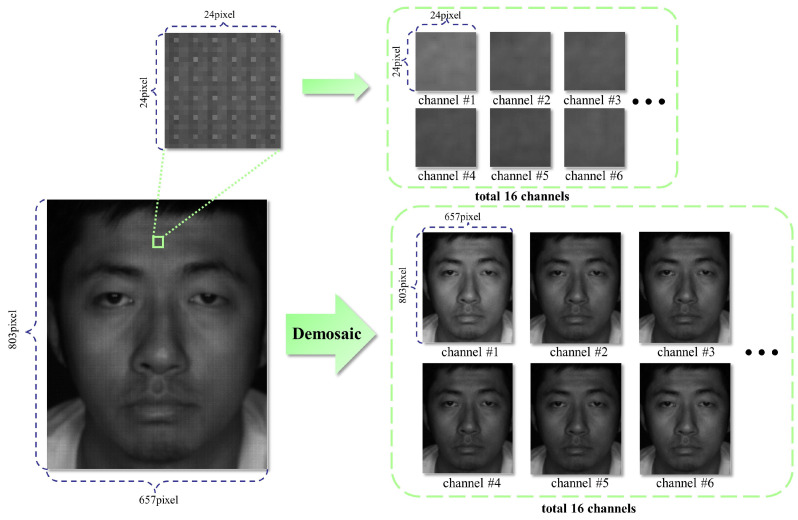
The effectiveness of the demosaicing algorithm. A 16-channel multi-spectral mosaic image is converted into 16 single-channel spectral images each maintaining the same size as the original mosaic image.

To ensure optimal accuracy, preprocessing of images and data is necessary. In order to guarantee the collection of identical spectral and light intensity information across different channels, we introduced a demosaicing algorithm specifically tailored to our system [38]. This demosaicing algorithm is able to compute the intensity estimated from the raw multi-spectral filter array (MSFA) image based on the correlation between each channel and the intensity [38]. The effectiveness of this algorithm is demonstrated in Figure 3, where a 16-channel multi-spectral mosaic image is converted into 16 single-channel spectral images, each maintaining the same size as the original mosaic image. Each channel is capable of restoring the DN values of other channels for one pixel. The demosaicing algorithm significantly reduces errors in spectral channel acquisition, ultimately enhancing overall spectral accuracy. Furthermore, the demosaicing algorithm mitigates resolution loss caused by different spectral channels. As a result, it improves the spatial resolution of spectral data in three dimensions. This is particularly beneficial for capturing fine details and nuances in the spectral information, leading to more precise and reliable results for subsequent analysis and reconstruction.

**Figure 4:**
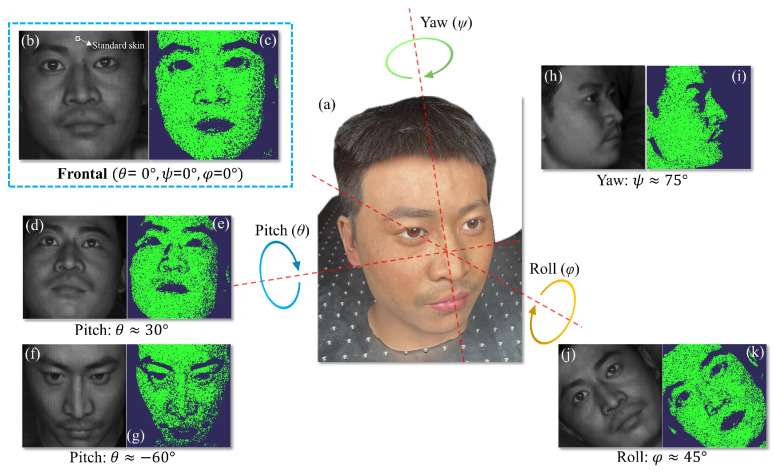
Multi-pose face recognition system. (**a**) Pose of face model measured by Euler angle (pitch, yaw and roll). (**b**) The mosaic image of frontal faces extracted by Dlib. The white frame in the mosaic image represents the standard value for the Euclidean distance algorithm. (**c**) Selected regions (green) of this face can be obtained through the algorithm for characterized spectra construction. The faces with maximum detectable Euler angles of (**d**,**f**,**h**,**j**) side faces can be captured by Dlib, as shown in the left images in each subplot, and the selected regions (**e**,**g**,**i**,**k**) can be correctly extracted by the Euclidean distance algorithm. The extracted regions are represented by the green area in the left map in each subplot. The maximum recognizable Euler angles are marked below each subplot of the side face, where the pitch angles range from −60° to 30°, the yaw angles range ±75°, and the roll angles range ±45°.

At present, the LFD method based on snapshot spectral imaging combines dictionary learning and compression sensing to achieve accurate spectral reconstruction [15,19,20]. However, it is extremely time-consuming. Through testing, it took several hours to reconstruct the spectra of the flat areas on a face. So, in their system, they have to select only a few spectral data points from fixed positions on the face to construct the datasets. When there are covers on the face or the face is not directly facing the system, the problems will be exposed. In contrast, our method has the capability of rapid and accurate reconstruction, which is able to deal with such problems more effectively. We have developed a multi-pose face recognition system comprising the Dlib [39] face detection algorithm and the Euclidean distance region selection algorithm. The Dlib algorithm is employed to locate and extract human facial regions from the entire image. Within a range of Euler angles [40], this algorithm consistently captures facial features in the mosaic raw image, accurately positioning the upper edge of the extraction to the forehead region. As long as there are no scars or obstructions on the forehead, the selected upper part of the facial regions will always be a flatter skin region. This region is then used as a standard region for extracting the 16-channel DN values as reference values. To determine the accuracy of the reconstruction, we computed the Euclidean distance [41] between the 16-channel DN values at each facial position and the reference values. Regions with a Euclidean distance below a specified threshold are considered selected reconstructed regions. This approach effectively identifies most flat areas of the face while excluding non-target areas such as eyebrows, mouth, and shadows. As demonstrated in Figure 4, we evaluated the multi-pose face recognition system across various facial Euler angles, determining the maximum detectable angle for each direction. The pitch angles range from −60° to 30°, the yaw angles range from ±75°, and the roll angles range ±45°. Within this range, the selected effective regions painted green accurately cover most of the flat areas for faces with different orientations, effectively eliminating regions that could potentially interfere with the spectral reconstruction accuracy. Unlike current spectral LFD technology limited to detecting fixed-angle faces and specified points of faces to extract spectra, our method operates across a broader Euler angle range, enabling a wider range of applications.

We extracted DN values pixel by pixel from the selected region, encompassing approximately 50,000 to 300,000 pixels per face, resulting in a huge dataset of DN values containing spectral information. Then, to mitigate differences in the intensity of reflected light, normalization was applied on the DN values, and they were subsequently reconstructed into an equivalent number of spectra. To further reduce time consumption and prevent the loss of weak spectral features around 500–650 nm, we intercept the 450–700 nm spectral band, which has a more obvious characteristic spectrum. The spectral data will be randomly arranged and compressed into a dataset that includes 2000 spectra by mean filtering. Compressing was able to shorten the training time, reduce the noise error, and may also sacrifice spectral features. The value 2000 was empirically obtained through multiple tests. It may not represent the optimal value, but minor adjustments to this parameter are unlikely to result in significant changes to the final result. We found that the data range from 50,000 to 300,000 spectra and are compressed by 20–200 times, with limited spectral line differences. To ensure that each sample had the same weight during training, 2000 was selected as the compression number.

**Figure 5:**
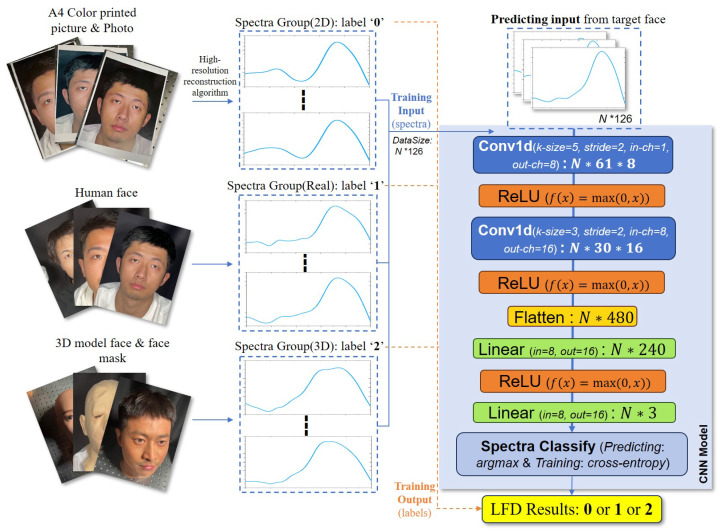
The architecture of the reflection spectrum database construction and the CNN model used. Images were reconstructed as spectral databases for each group, and the CNN model was trained on half of the data. The *N* values represent the batch size of model training. The reflection spectra input into the model can be classified as one of the groups named “2D”, “Real”, and “3D”, and were numbered 0, 1, 2, respectively, which represents the group sequence number. Spectral data served as the training input, and the label number served as the training output. The CNN network model is shown in the light blue frame on the right side of figure. During network training, we used cross-entropy loss to measure prediction errors and adopted the Adadelta optimization method for parameter updates. And in the prediction phase, we apply the argmax function to classify samples based on the predicted probability distribution. After repeated testing, the optimal network structure was marked on the model figure. Within the Conv1d modules, ‘k-size’ denotes the kernel size, and the ‘in-ch’ and ‘out-ch’ represent in-channels and out-channels, respectively. And within the linear modules, ‘in’ and ‘out’ represent in features and out features, respectively.

The samples we selected include real faces, A4 color printed paper, photos, 3D model faces, and face masks. They are divided into three groups, real face, 2D fake face, and 3D fake face, and data in the same group have the same label. Fifty percent of the samples in each group were utilized to train the neural network model and twenty percent of the samples were employed for validating the model; the reminder were used validating the LFD system. The architecture of the reflection spectra database construction and CNN model we designed are shown in Figure 5. Thanks to the large amount of spectral data we extracted, convolutional layers in deep neural networks can better extract dominant and latent spectral features of faces than machine learning [42]. The datasets for each “face”, along with their group labels, had two-thirds of the data designated as the training set for the network, with the remaining one-third serving as the validation set, which is excluded from the network training process. After passing through the network, a spectral curve is downsampled into three eigenvalues that represent the probabilities of belonging to different groups (the number of eigenvalues is equal to the number of target groups). We use the cross-entropy loss function to calculate the loss for the passing data, and the training and validation loss plot will be used for model quality evaluation and parameter adjustment.

After the training was completed, the target face reconstruction spectra were input into the trained model, which produced the LFD results. Depending on the group label, the argmax module outputs within the group sequence numbers 0, 1, 2 based on the maximum probability of eigenvalues. So, in the test stage, the other half of the sample “faces” were used to assess the performance of the system. The quality of system performance can only be validated when the test set data demonstrates high accuracy.

## 4. Experiment and Results

### 4.1. Experimental Setup

**Figure 6:**
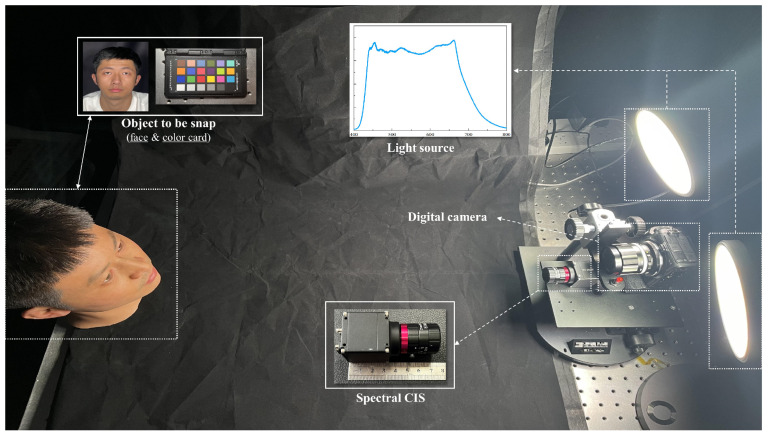
The experimental setup of the liveness detection system. A color digital camera was placed adjacent to the sensor. The full-spectrum lamps were set on each side of the spectral CIS.

The experimental setup is visually depicted in Figure 6, where we chose two full-spectrum lamps (LT01, Thinkplus) as the light sources. Our system employed active light sources to provide illumination; the lamps were carefully selected to ensure uniform illumination with a flat spectrum across the target band. A color digital camera was placed adjacent to the sensor to capture images that closely resemble real faces. The 24-color colorchecker (Calibrite) mentioned in the figure is used to verify the spectral reconstruction effect. To expand the range of facial detection, our sample selection included real faces, 3D simulation models, masks, photos, and A4 color printed pictures. These samples were categorized into three groups of real faces, 3D fake faces, and 2D fake faces. The spectra of the measured samples, along with their corresponding group labels, were input into the CNN for LFD model training.

To ensure consistent and accurate results, it was crucial to maintain a constant relative position between the light sources and different samples. In order to achieve that the incident light on the sample surfaces remained similar throughout the experiment, we utilized a spectral CIS along with a digital camera that did not undergo white-balance correction. This allowed us to capture images of the target in the exact same lighting environment. Following the capture of face images using the digital camera, we printed these images on both photos and papers. These printed images served as additional face samples, mimicking the appearance, colors, and shadows of real faces. By utilizing our snapshot spectral CIS, we obtained multiple sets of mosaic images. These mosaic images were then used as raw image data for subsequent processing and analysis in our study.

We recruited a total of 28 Asian volunteers, obtaining real human face samples from individuals of different genders and ages ranging from 21 to 55 years old, which represents the demographic most frequently utilizing the LFD system. We took five sets of data for each face from different Euler angles to expand the dataset and obtained a total of 140 images. The 2D fake face sample acquisition method mentioned above generates an equivalent quantity of A4 color printed paper and photo samples and obtained a total of 140 images. We meticulously prepared eight face masks and six 3D face models made of silicone and synthetic resin, whose colors are more proximate to the skin tones of East Asians and Caucasians, with each sample obtaining five images from different Euler angles and obtaining a total of 40 images and 30 images, respectively. Half of the samples within each sample group are utilized for training, while the remainder are employed for validation and LFD accuracy testing.

### 4.2. Results

To begin with, the performance of the spectral CIS detector and the reconstruction algorithm under controlled experimental conditions were evaluated.

We built a reference spectrum acquisition system using an optical fiber spectrometer (FX2000-RD, Ideaoptics), extended fibers, and fiber-optic focusing mirrors, calibrated by standard light sources. Given that the spectrum collected by the spectrometer represents the integral value across a specific region, which is typically larger than the area of a single sensor pixel, we aimed to establish the correlation between the integral spectrum obtained by the reference spectrum acquisition system and the reconstructed spectrum of the “micro-spectrometer” within the sensor. To achieve this, we employed a 24-color standard color card as the detection target, ensuring a uniform color distribution within each color block. Treating the integral spectrum of the color block region as equivalent to the spectrum of any single point, we utilized the reference spectrum acquisition system to gather reference spectrum data from the standard color card. Subsequently, we compared this spectrum with the single pixel reconstruction spectrum (pixels = 1 × 1) and the regionally averaged reconstruction spectrum (pixels = 20 × 20) collected by the LFD system under identical experimental conditions.

**Figure 7:**
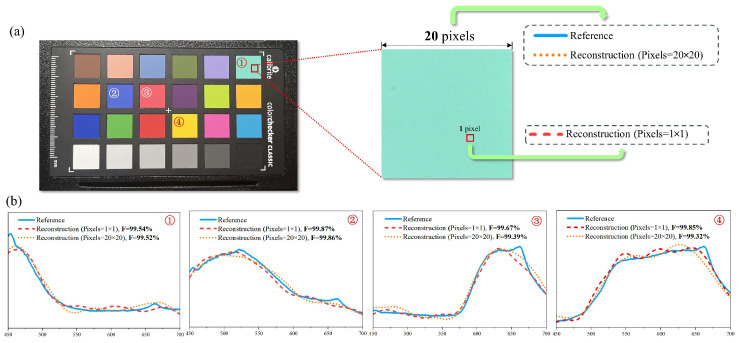
The 24-color standard color card spectral reconstruction results. (**a**) Spectrum acquisition position of color card. The reference spectrum and regionally averaged reconstruction spectra collected from a 20 × 20-pixel region and single-pixel reconstruction spectra obtained from single pixels in 20 × 20-pixel region. (**b**) Subfigure (1–4) shows the comparison between the reference spectra (blue), regionally averaged reconstruction spectra (red), and single-pixel reconstruction spectra (orange) of four different colors from the standard color card of pale green, Han blue, watermelon pink, and yellow, respectively (mark (1–4) shown in (**a**)). The **F** values representing the fidelity of regionally averaged reconstruction spectra were 99.52%, 99.89%, 99.39%, and 99.32%, respectively, and the fidelities of single-pixel reconstruction spectra were 99.54%, 99.87%, 99.67%, and 99.85%, respectively.

In order to quantify the similarity between the reference spectra and the reconstructed spectra, we introduced the concept of fidelity [43]:

$$F\left(X,Y\right)={\left(\sum_{m}\sqrt{p_{m}q_{m}}\right)}^{2}$$

where *X* and *Y* represent the normalized reference and reconstructed spectra, respectively, and and denote the corresponding intensities at the *m*th wavelength sampling point. As shown in Figure 7, after processing by the demosaicing algorithm, the fidelities of regionally averaged reconstruction spectra of four different color were 99.52%, 99.89%, 99.39% and 99.32% for pale green, watermelon pink, Han blue, and yellow, respectively. The fidelities of single-pixel reconstruction spectra were 99.54%, 99.87%, 99.67%, and 99.85%, respectively, which were close to the fidelities of averaged data. The 24-color standard color card was captured in one image by the spectral CIS, so it can be inferred that there were minor differences in different pixels. The fidelity results indicate that our spectral imaging system is capable of accurately reconstructing the reflection spectrum.

**Figure 8:**
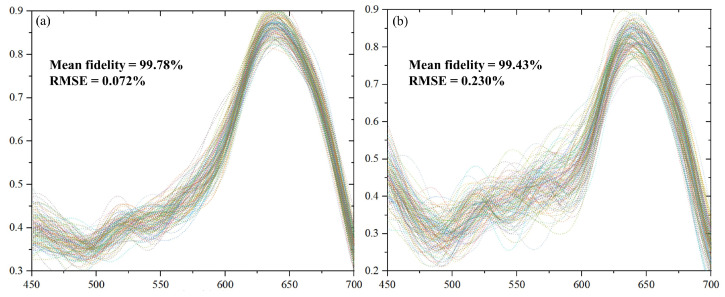
The effect of demosaicing algorithm on the spectral data distribution with (**a**) demosaicing-on and (**b**) demosaicing-off mode. The 200 spectra in each plot were randomly extracted from the reconstructed database. Their mean fidelities were 99.78% and 99.43%, and their RMSEs were 0.072% and 0.230%.

Next, we investigated the optimization effect of the demosaicing algorithm on the reconstruction process. We took the original demosaicing algorithm, namely reordering the image data into a three-dimensional matrix, as a reference. As shown in Figure 8, we compared the reconstruction effect with or without the demosaicing algorithm; each plot consists of 200 spectra randomly extracted from reconstructed database. The reconstructed spectra with the demosaicing algorithm have higher fidelity and less root mean square error (RMSE). Obviously, the demosaicing algorithm effectively enhances the fidelity and stability of spectral reconstruction, reducing the disturbances caused by detection misalignment.

**Figure 9:**
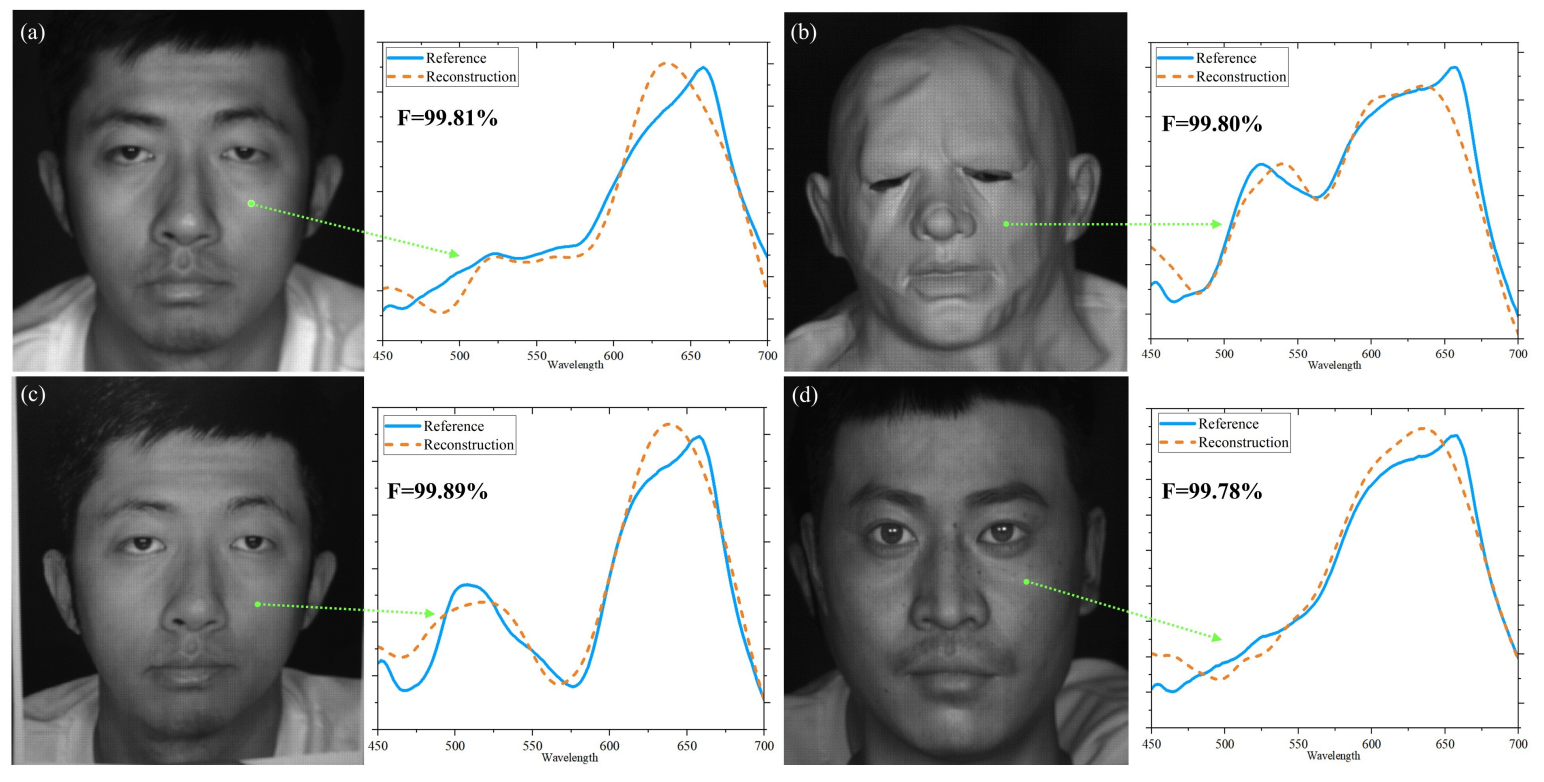
The reconstructed spectra (orange) and their reference spectra (blue) of different “face” samples for (**a**) real face, (**b**) face mask, (**c**) color printed paper, and (**d**) 3D model face. The **F** values representing the fidelity of each spectrum were, respectively, 99.81%, 99.80%, 99.89%, and 99.78%.

Afterwards, we performed tests to assess the fidelity of spectral reconstruction on various ‘face’ samples in the same experimental conditions, and the detailed results are illustrated in Figure 8. The samples, as well as their contrast results in Figure 9a–d, came from a real face, a face mask, color printed paper, and a 3D model face, respectively. The calculated fidelities signed on each plot were 99.81%, 99.80%, 99.89%, and 99.78%, respectively. With the help of the demosaicing algorithm, our system can reconstruct the reflectance spectra of sample faces accurately. The high-fidelity values also demonstrate the accuracy and reliability of our spectral imaging system on different types of ‘face’ samples.

**Figure 10:**
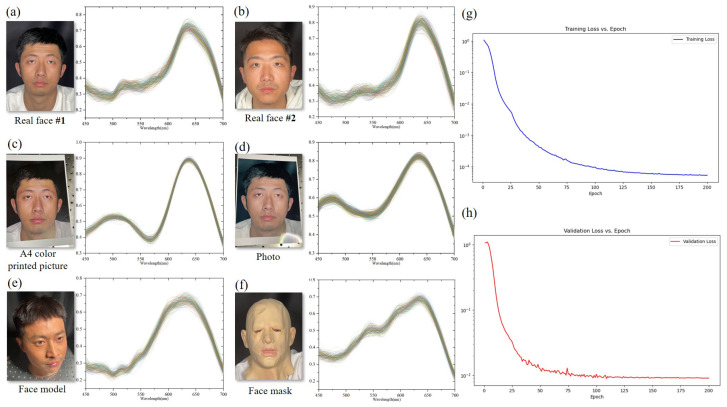
The reconstructed spectra of different “face” samples for (**a**) real face of testee #1, (**b**) real face of testee #2, (**c**) 3D model face, (**d**) A4 color printed paper, (**e**) photo, and (**f**) face mask. Respectively, the 300 spectra in each plot were randomly extracted from the reconstructed database. Losses of (**g**) training and (**h**) validation is continuously convergent during gradient descent.

To build the database, we needed to reconstruct the spectra from each point of the selected ‘face’ region, resulting in approximately 50,000 to 300,000 reflected spectra. It is worth mentioning that reconstructing 300,000 reflected spectra of a single face required approximately 0.3 s. The advantage of fast-processing meets our requirements for minimal time consumption, especially considering the large dataset size of 300,000 spectra, making the spectral imaging system highly efficient for handling such a huge dataset. According to the normalization algorithm and the mean compression algorithm mentioned above, these spectra were compressed into 2000 spectra and organized into a dataset. We used this dataset to train and evaluate the face classifier using the Adadelta optimizer. Apart from this, based on the ablation study detailed in Table S1 (See Supplementary Material for more details), the batch size was 2048, the number of iterations was 200, and the initial learning rate was 0.0004, which decays by half every 25 epochs. During model training, the minimum training loss and the validation loss are continuously recorded, and a model checkpoint is saved whenever the minimum validation loss is updated. If the minimum value of either the training loss or validation loss has not been updated for 10 consecutive epochs and the epoch value exceeds 10, it is deemed that further training may lead to overfitting. Consequently, the model checkpoint will no longer be saved. Meanwhile, training continues until the specified number of iterations is completed to obtain the full loss curve, which can be used for further model optimization. Figure 10 presents the typical reconstructed “face” spectra from datasets and the training and validation loss vs. epoch curve. The sample images obtained from the LFD system included different real faces, 3D model faces, masks of face, A4 color printed paper, and photos. Each image in the figure contains 300 spectra randomly extracted from the reconstructed database, representing the data that would be incorporated into the dataset. Significant differences in the different types of ‘face’ spectra could be observed, with distinct peak–valley shapes observed in the spectral curves, the characteristic spectra of real human faces have been accurately extracted (See Supplementary Material for more details). This pattern is consistent across multiple experiments. Multiple sets of samples were obtained for the study, and a specific subset was assigned for training purposes. To assess the system’s performance, the dataset was divided into training and validation sets, with two-thirds of the spectral data allocated for training and the remaining one-third reserved for testing. The loss function of training and validation can correctly and continuously demonstrate that the CNN-based LFD model was successfully trained.

**Figure 11:**
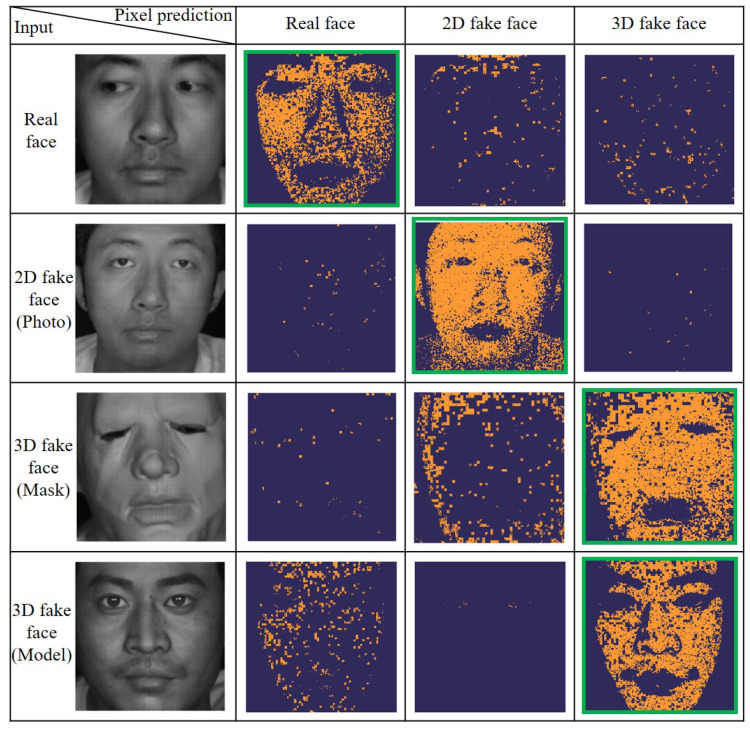
Pixel-level prediction maps and corresponding input image liveness detection of faces. The orange points represent predicted types of corresponding targets. The images enclosed within the green frames represent the majority prediction.

To demonstrate the ability of the LFD method to accurately predict material groups for samples, a pixel-level liveness detection test system was developed based on our LFD system. It has been demonstrated previously that the reconstructed spectrum of a single pixel in a flat region approximates the mean spectrum of nearby region. So after selecting regions based on Euclidean distance and point-by-point spectral reconstruction, each cell within the image is represented by the mean spectrum derived from the selected region within each 10 × 10-pixel grid to save computation and data storage costs. The spectrum of each cell was sequentially input into the model to predict the material types of the ‘face’ region. The predicted maps of different types of sample ‘faces’ are shown in Figure 11. In this figure, the header columns correspond to different types of input samples, including real faces, photos, masks, and models. The header rows represent different types of pixel prediction results, including real faces, 2D fake faces, and 3D fake faces. The orange points represent the predicted types of corresponding targets. The percentage of orange points on the map indicates the predicted probability of the corresponding targets. The images within the green frames describe the majority prediction for each input sample, with the proportions of 83.31%, 97.36%, 79.11%, and 79.85% for real faces, photos, face masks, and face models, respectively. Obviously, most of the predictions are consistent with the types of input samples, which demonstrates the ability to conduct pixel-level liveness detection. The evaluation of generalization ability (See Supplementary Material for more detail) further demonstrated that, our approach remains effective in complex scenarios involving interfering light sources and facial occlusions. It indicates that our method is not confined to live face detection; should we successfully extract the target pixels, it will be able to identify materials with distinct spectral features. It presents significant potential for applications in near-range material classification and anti-fraud strategies.

In an effort to further minimize time consumption, the spectra of the target ‘face’ were compressed into a set of 2000 spectra, following the same procedure used for dataset construction. The samples that were not involved in the network training were utilized as validation samples for LFD and output the predicted labels of each reconstruction spectrum. The proportion of each predicted label indicates the prediction probability of each ‘face’ type, resulting in the detection results detailed in Table 1. Our algorithm was decomposed and analyzed, and certain data processing algorithms were optimized by @jit decorator, significantly reducing the prediction time. We detected 64 samples and recorded the time consumed. Due to variations in the size of the reconstructed data for each sample, the prediction time per sample ranged from 1.0333 to 2.356 s, with an average prediction time of 1.621 s. Among them, data preprocessing and Dlib face location consume an average of 0.509 s, the demosaicing algorithm consumes 0.654 s, flat area screening consumes 0.047 s, spectral reconstruction and data compression consume 0.407 s, and the final prediction consumes 0.004 s. It is noteworthy that the number of validation samples significantly exceeded the number of training samples. To further diversify the validation experiment, we incorporated additional photos of faces downloaded from the Internet. The LFD system exhibited an accuracy exceeding 98% under consistent light source conditions, with consistent results across various sample groups. It demonstrated robust performance across diverse samples, showcasing its versatility and commonality. The variability introduced by the data compression strategy results in a slight discrepancy between datasets generated from the same plot. However, the observed accuracy change attributed to these differences is not more than 1% based on our tests. In addition, we reproduced images of model faces and masks on photos and paper, achieving accurate classification into the correct groups.

## 5. Conclusions

We designed a novel LFD method utilizing a compact spectral CIS and built a system based on this method. Our system enables simultaneous capture of both spatial and spectral data using a single sensor in one shot, making it fully compatible with real-time dynamic face identification usage scenarios. Significantly, our system exhibits superior performance in comparison to traditional spectral LFD systems, enabling point-by-point spectral reconstruction of the flat facial skin area within a short frame, which eliminates the need for time-consuming scanning procedures and face orientation requirements. Owing to the spectral modulation utilizing the Tikhonov spectral reconstruction algorithm, our system achieves a precise spectral reconstruction fidelity of over 99% for the color checker and different types of ‘face’ samples. To acquire accurate initial DN values, we employed a demosaicing algorithm, which reduced the RMSE to one-third of its original value. We selected skin regions based on Euclidean distance and compressed their reconstruction spectra into a huge database. A CNN model was trained on this database, which was capable of predicting different types of ‘faces’ with more precision (98%), and according to a series of evaluations, the system’s detection time consistently remained below one second and has initial real-time capability. Meanwhile, a pixel-level liveness detection test system was also developed, and the LFD experiment shows good agreement with theoretical results, which proves that the method has the potential to be applied to other fields. The details of the comparison of LFD systems are shown in Table 2, including RGB cameras (SIFT and others) [24,25,26], traditional multi-dimensional cameras (RGBD and others) [27], advanced multi-dimensional cameras (SWIR and others) [28,29,30], traditional HSI [11,12], snapshot HSI [15] and ours.

There are several aspects of our research that still require additional efforts for optimization. Firstly, constrained by the scale of the study and the privacy of the data we have collected, we have not conducted large-scale volunteer recruitment as of yet. We lacked real-life data of teenagers, people over 60 years of age, dermatology patients, and non-Asian ethnic groups, which makes our research results regionally specific. Once our findings are acknowledged, we will expand the scope of volunteer recruitment in the future and attempt to develop more detection classifications underpinned by the theory of biomedical spectroscopy. Meanwhile, our system code compilation relies on a Python platform that boasts excellent development efficiency and a superior software ecosystem; however, it suffers from a disadvantage in terms of computing speed. It can be predicted that the incorporation of hardware acceleration methods, such as FPGA [44] or C++ programming, would significantly improve the processing speed and overall recognition time of our LFD system.

In conclusion, compared to existing LFD systems, our system stands out as a cost-effective, compact, and versatile solution. Its compatibility allows for seamless integration into various imaging platforms, such as an access control system or mobile phones, making it highly convenient for the widespread adoption of this technology. This innovative approach effectively mitigates the barriers to adopting LFD systems based on spectral imaging, providing an alternative solution for accurate, highly integrated video LFD applications.

## Figures and Tables

**Table 1:** Accuracy evaluation results of live face detection.

	Accuracy (%)	RMSE (%)
Real face	98.89	4.33
Color printed paper	99.52	0.39
Photo	99.97	0.07
3D model face	99.15	0.97
Face mask	99.71	0.44
Color printed paper (Internet)	99.29	0.96

**Table 2:** Comparison of live face detection systems.

	3D LFD	Integration	No Scanning	Illum.-Indep.	Covers	Multipose
RGB camera	✗	✓	✓	✓	✗	✓
Multi-dimensional	✗	✗	✓	✗	✓	✓
Advanced camera	✓	✗	✓	✗	✓	✓
HSI	✓	✗	✗	✓	✓	✓
Snapshot HSI	✓	✓	✓	✓	✗	✗
Ours	✓	✓	✓	✗	✓	✓

## Data Availability

Data underlying the results presented in this paper are not publicly available at this time but may be obtained from the authors upon reasonable request.

## Supplementary Materials

The following supporting information can be downloaded at: https://www.mdpi.com/article/10.3390/s25030952/s1, The supplementary material provides additional details on the experimental setup, derivations, and extra results. The document is available as a separate file.
