# Supplementary material for: Accurate Real-Time Live Face Detection Using Snapshot Spectral Imaging Method
Source: Sensors (Basel). 2025 Feb 5;25(3):952. doi: 10.3390/s25030952 (PMC11820336; doi:10.3390/s25030952)
Supplement: Supplementary file 1 [file sensors-25-00952-s001.zip › sensors-3340321-supplementary.pdf]

1. Ablation Study

We conduct a series of model evaluations by adjusting hyperparameters or activation functions, and the results are shown in Talbe S1. Among them, hyperparameters include learning rate and batch size. The algorithm can be stopped early when overfitting occurs, so that adjusting the number of iterations will not have a significant impact on the results unless the number of iterations is too less. Ultimately, through the adjustment of the learning rate and batch size, we obtained a set of hyperparameters that offered a balanced detection performance for different samples.

Meanwhile, hyperparameters include Relu, Sigmoid and Softmax. According to multiple tests, the test using the Sigmoid activation function can converge Successfully. However, no matter how the hyperparameters were adjusted, the detection results were always bad. The test using the Sigmoid activation function cannot effectively converge under different hyperparameter settings.

Given that the network training itself possessed randomness, the final detection results obtained from the same hyperparameters may fluctuate within a certain range. Therefore, the samples of the best learning rate for each batch size (exp.1, exp.5, exp.8) in actual results are extremely close, and all of them can perform the detection task well.

Table S1. The result of different hyperparameters or activation functions on model performance.

Exp.	Activation functions	Learning rate	Batch size	Convergence	Acc. of Real face (%)	Acc. of 3D fake face (%)	Acc. of 2D fake face (%)
1	Relu	0.0004	2048	√	98.89	99.69	99.74
2	Relu	0.0008	2048	√	97.66	99.84	99.18
3	Relu	0.0002	2048	√	95.16	99.85	96.13
4	Relu	0.0002	1024	√	96.21	98.82	96.58
5	Relu	0.0004	1024	√	97.08	99.91	98.75
6	Relu	0.0006	1024	√	98.16	98.30	96.66
7	Relu	0.0004	4096	√	97.48	98.70	97.28
8	Relu	0.0008	4096	√	98.18	99.84	99.78
9	Relu	0.0012	4096	√	92.67	98.38	96.59
10	Sigmoid	0.0004	2048	√	59.06	73.62	66.17
11	Softmax	0.0004	2048	×	×	×	×

2. Spectral feature extraction

To reveal the learned feature patterns of the model, we have plotted the feature heat-maps of various samples based on 1dGrad-CAM. It generated feature weights of length 30 based on the gradient features of last convolutional layers, meaning that every 4.2 spectral sampling points correspond to one feature weight. We normalized the feature weights, with the color temperature in the heatmap being lower (closer to red) indicating a higher weight and higher usage frequency, while a higher color temperature (closer to blue) indicates a lower weight and lower usage frequency.

We presented feature heat-maps for the facial spectrum of real face, color-printed pictures, face models and face mask in Figure S1. It shows that real faces and 3D fake faces are primarily classified based on spectra around 480 – 580 nm. In contrast, the reflection spectrum of 2D fake faces comes from colored ink reflections, which exhibit more pronounced differences from real human faces, with higher spectral features in the 600 – 650 nm range. This suggests that the feature spectrum we rely on is similar to the theoretical human face spectral range, highlighting the close connection between model learning and spectral features.

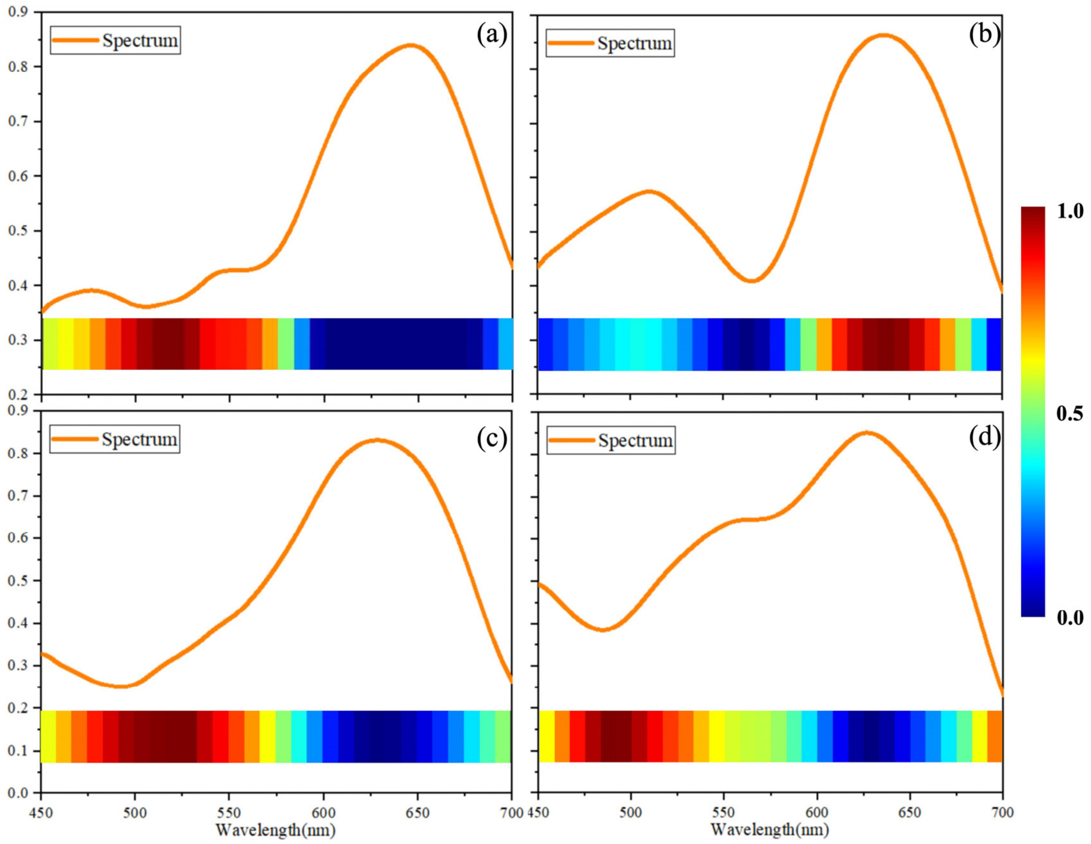

Figure S1. Detected spectrum and their feature heat-maps based on 1dGrad-CAM obtained from (a) real face, (b) color-printed pictures, (c) face models and (d) face mask. The feature weights were normalized and shown as the heat-maps, the color temperature in the heatmap being lower (closer to red) indicating a higher weight, while a higher color temperature (closer to blue) indicates a lower weight.

3. Generalization ability

Since the system employs an active light source, the facial region receives significantly higher irradiance compared to the background. Meanwhile, the multi-pose face recognition system retains only the information from flat facial regions, meaning background environmental information typically does not directly affect the training data. To demonstrate this, as shown in Figure S2 (a), we prepared a test chart with rich colors as the background for subsequent testing. In the captured images, the background was clearly imaged behind the "face," acting as background interference for LFD.

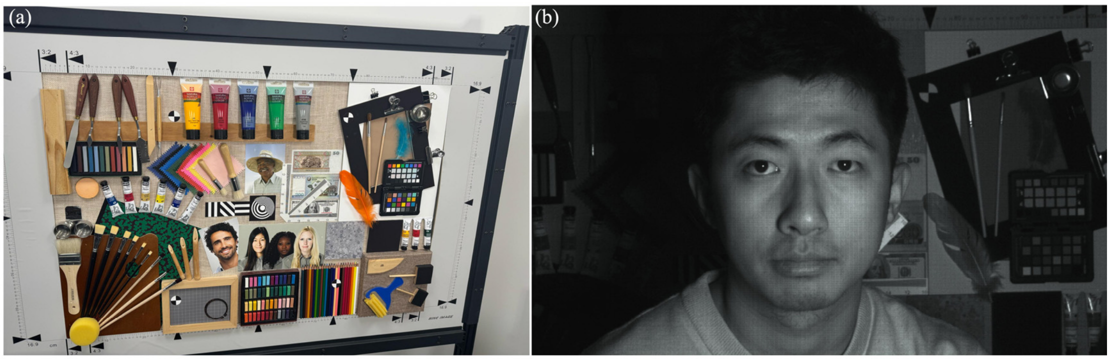

Figure S2. (a) Colorful background with various objects and (b) example of a complete mosaic image captured by the sensor.

To fully demonstrate the generalization capability of our system, we selected a halogen lamp, whose spectral lines differ significantly from our active light source, as an interfering light source. The active light source and the interfering light source simultaneously illuminated the samples. The interfering light source was positioned at a distance 1.5 times that between the active light source and the sample, at an angle of approximately 30 degrees to the horizontal direction of the ‘face’ to simulate common levels of interference intensity. We conducted liveness detection on previously challenging samples, including real human faces, silicone model and latex model, under conditions of various facial expressions and facial occlusions. Due to the number of supplemental experiments were much less initial tests in main text, we employed pixel-level liveness detection to evaluate classification performance. It tested whether data could be correctly extracted and accurately classified for untrained samples involving occlusions or unusual expressions.

As shown in Figure S3, the accuracy of detecting the real face, real face with different expressions, latex model, silicone model, real face with occlusion and silicone model with occlusion were 80.07%, 79.23%, 74.43%, 74.42%, 84.26% and 75.40% respectively. Similar to the results presented in the main text, most material regions were correctly classified, and facial occlusion areas were successfully excluded, interference light sources, different facial expressions, additional backgrounds, and minor facial occlusions will not significantly interfere with the

detection accuracy. It demonstrates the robust generalization capability of the LFD system.

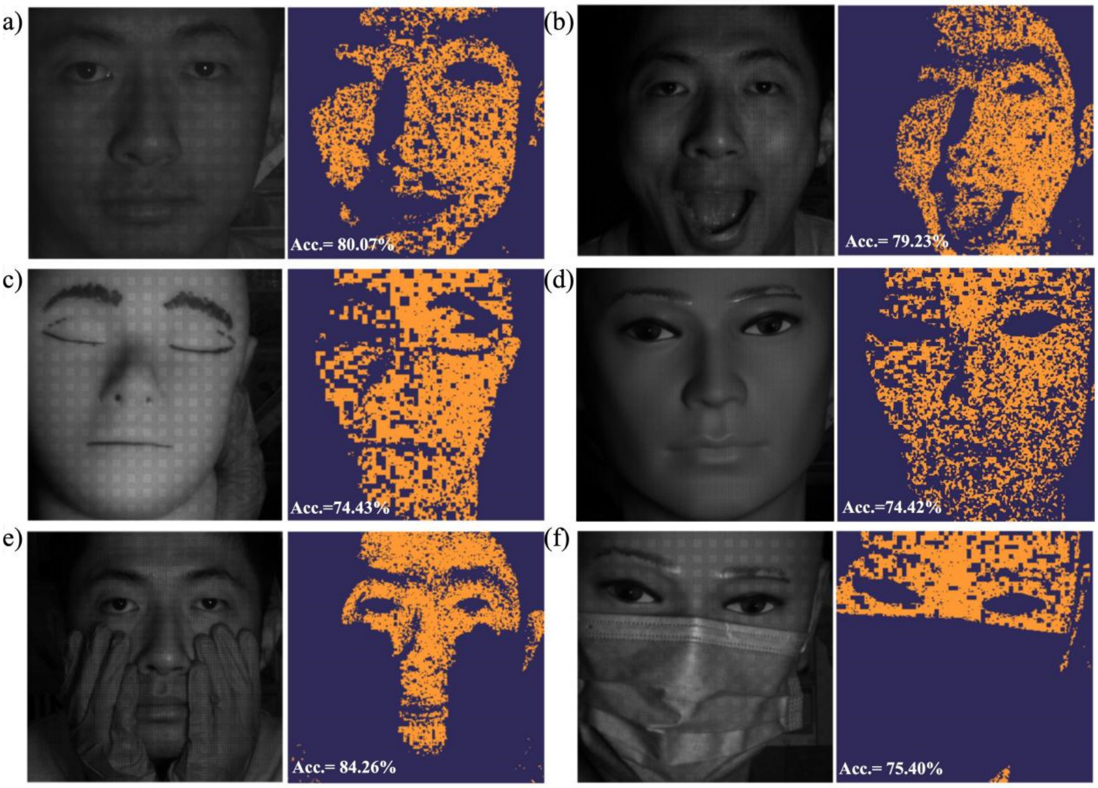

Figure S3. Under active light source and halogen lamp illumination, the results of pixel-level liveness detection for different samples. (a) Real face. (b) Real face with different expressions. (c) Latex model. (d) Silicone model. (e) Real face with occlusion. (f) Silicone model with occlusion. The orange areas represent regions identified as the correct target material, while the Acc. in figure denotes the recognition accuracy.
